# Optomechanical Processing of Silver Colloids: New Generation of Nanoparticle–Polymer Composites with Bactericidal Effect

**DOI:** 10.3390/ijms22010312

**Published:** 2020-12-30

**Authors:** Jakub Siegel, Markéta Kaimlová, Barbora Vyhnálková, Andrii Trelin, Oleksiy Lyutakov, Petr Slepička, Václav Švorčík, Martin Veselý, Barbora Vokatá, Petr Malinský, Miroslav Šlouf, Pavel Hasal, Tomáš Hubáček

**Affiliations:** 1 Department of Solid State Engineering, University of Chemistry and Technology Prague, 166 28 Prague, Czech Republic; polivkoa@vscht.cz (M.K.); barbora.vahnalkova@vscht.cz (B.V.); trelinn@vscht.cz (A.T.); lyutakoo@vscht.cz (O.L.); petr.slepicka@vscht.cz (P.S.); Vaclav.svorcik@vscht.cz (V.Š.); 2 Department of Organic Technology, University of Chemistry and Technology Prague, 166 28 Prague, Czech Republic; veselyr@vscht.cz; 3 Department of Microbiology, University of Chemistry and Technology Prague, 166 28 Prague, Czech Republic; vokataa@vscht.cz; 4 Department of Physics, Faculty of Science, University of Jan Evangelista in Ústí nad Labem, 400 03 Usti nad Labem, Czech Republic; malinsky@ujf.cas.cz; 5 Institute of Macromolecular Chemistry, Academy of Sciences of the Czech Republic, Heyrovského nám. 2, 162 06 Prague, Czech Republic; slouf@imc.cas.cz; 6 Department of Chemical Engineering, University of Chemistry and Technology Prague, 166 28 Prague, Czech Republic; hasalp@vscht.cz; 7 Biology Centre of the Czech Academy of Sciences, SoWa National Research Infrastructure, Na Sádkách 7, 370 05 České Budejovice, Czech Republic; hubacektom@gmail.com

**Keywords:** silver nanoparticles, polymer, excimer laser, immobilization, surface characterization, antibacterial activity

## Abstract

The properties of materials at the nanoscale open up new methodologies for engineering prospective materials usable in high-end applications. The preparation of composite materials with a high content of an active component on their surface is one of the current challenges of materials engineering. This concept significantly increases the efficiency of heterogeneous processes moderated by the active component, typically in biological applications, catalysis, or drug delivery. Here we introduce a general approach, based on laser-induced optomechanical processing of silver colloids, for the preparation of polymer surfaces highly enriched with silver nanoparticles (AgNPs). As a result, the AgNPs are firmly immobilized in a thin surface layer without the use of any other chemical mediators. We have shown that our approach is applicable to a broad spectrum of polymer foils, regardless of whether they absorb laser light or not. However, if the laser radiation is absorbed, it is possible to transform smooth surface morphology of the polymer into a roughened one with a higher specific surface area. Analyses of the release of silver from the polymer surface together with antibacterial tests suggested that these materials could be suitable candidates in the fight against nosocomial infections and could inhibit the formation of biofilms with a long-term effect.

## 1. Introduction

Nanoscale-sized metal particles are indispensable in numerous industrially important applications such as catalysis [1,2,3], healthcare (targeted cancer therapy [4], antibacterial treatment of medical devices [5,6], biochips [7], drug delivery [8]), cosmetics [9], and the textile industry [10,11]. The typical characteristics of materials exploitable in these applications are the large interaction area between active components (nanoparticles (NPs)) and the environment with simultaneous minimal leakage or targeted release of those active components from the reaction/interaction area into surroundings. In addition, NPs must withstand severe reaction conditions without the tendency to coalesce, which may significantly reduce the efficiency of every required process. In this regard, immobilization techniques onto carrier substrates seem to be effective tools which, together with a sufficiently high surface concentration of active particles, guarantee the above-mentioned requirements and maintain a sufficient lifetime of these composite materials in NP-mediated processes. Thus, suitable immobilization processes of the active particles to the catalyst carriers [3], medical devices [5], or textiles [10], preventing them from being released into the environment, are intensively sought. A strong nanoparticle-carrier anchorage extends both the efficiency and lifetime of such high-end products and prevents environmental contamination. In addition, it effectively blocks the flocculation of metal particles, which often occurs in simple colloids.

Many efforts have been undertaken in the field of particle anchoring to various types of materials. Conventional approaches nowadays include (i) anchoring of NPs by means of covalent chemical bonds [12,13], (ii) simple physisorption [14], (iii) particle dispersion in the entire carrier volume [15], or (iv) implantation [16]. Besides implantation, which belongs to low through-pass methods, and physisorption, characterized by low binding force, other methods are usable; however, fine applications especially in healthcare or catalysis may be sensitive to the chemical bonding interlayer [17]. Easily applicable particle dispersion in the entire carrier volume may significantly reduce the concentration of active particles in every surface area [18], which disproportionately increases costs and reduces the efficiency of particle-mediated processes.

Considering this, optomechanical manipulation with objects whose dimensions are orders of magnitude smaller compared to the wavelength of excitation light seems to be a promising alternative which opens up new possibilities in the design of prospective composite materials combining nanoparticles and polymers. Such materials excel in all important aspects, such as absence of a chemical bonding interlayer, presence of active particles only at the surface area of supporting material, and high bonding strength between active particles and carriers, preventing their release into surrounding environments [19]. Current studies in the field of trapping and manipulation with objects on the microscale, also including organic cells, are widely being published, but those dealing with manipulation with nanoparticles, especially metal ones, are still rare. In terms of their optical and electric properties, metal nanoparticles are more challenging for optical-induced manipulation since they offer ample new phenomena to explore, such as plasmon resonance [20]. The resonance and size effects have an impact on induced optical forces which make nanoparticle manipulation possible, and which differ from those forces common on larger micron-sized objects. In this regard, we have recently introduced a novel technique for immobilization of silver nanoparticles (AgNPs) by the action of polarized laser light, allowing the preparation of Ag nanoparticle arrays in the thin surface layer of polyethylenterephthalate film [19]. Our approach is based on collective optomechanical manipulation with silver nanoparticles by light, whose wavelength is pretty close to the local maxima of localized surface plasmon (LSP) excited in AgNPs.

In this paper, we introduce the mechanism behind this phenomenon together with the possibility to tailor resulting surface morphology of those polymers which absorb the light, by using proper laser fluence. By the fluence variation one can control resulting surface area morphology, which is essential in the vast majority of high-end applications. Finally, the creation of AgNP antimicrobial coatings is demonstrated on PET, whose antibacterial potency has been tested by drop plate method on selected Gram-positive and Gram-negative bacteria. The versatility of the proposed method lies in the possibility of immobilizing silver nanoparticles on the surface of a broad spectrum of polymers, regardless of whether they (polymer substrates) absorb radiation of a given wavelength or not.

## 2. Theoretical Background

The existence of radiation pressure was already being postulated in the beginning of the 17th century by Johannes Kepler (*De Cometis, 1617*), when explaining the twisting of a comet’s tail in a direction away from the sun. Along with further developments in electromagnetism, optical forces nowadays are categorized as conservative (gradient force) and non-conservative ones (scattering and absorption force) [21]. This concept enables us to identify two resulting actions of such forces, as the (i) gradient force is responsible for optical trapping (pulling particle into the beam, opposite to the direction of its propagation), while (ii) scattering force is responsible for particle transportation in the direction of the light propagation. Optical trapping and manipulation with particles under action of “gradient forces” are usually conducted in highly focused laser beams, where the light exerts force through polarization and wavelength-dependent transfer of angular photon momentum, resulting in trapping objects localized closed to an area of a focus point [22]. On the other hand, when the laser beam is polarized and collimated, incoming light exerts force through polarization and wavelength-dependent transfer of linear photon momentum, resulting in the scattering force pushing particles in the direction of light propagation. In that case, the object is usually pushed by electromagnetic waves due to the fact that the scattered radiation cannot be unidirectional [23]. In an analogy with electrostatics, small particles develop an electric dipole moment in response to the light’s electric field. The induced dipole is then drawn by field intensity gradients, which compete with radiation pressure due to momentum transferred from the photons in the beam. In free space, radiation pressure is proportional to the Poynting vector, which determines the direction and magnitude of the momentum flow [24,25]. Physically, the Poynting vector expresses the law of conservation of light momentum after its interaction with particles on which scattering occurs. Moreover, due to strong absorbance of metallic nanoparticles at wavelengths close to the maxima of localized surface plasmon resonance, light absorption leads to a temperature rise of the particle and the liquid surrounding it [26]. These two phenomena e.g., combination of scattering force with simultaneous heating of metal nanoparticles due to light absorption, are behind the principle of particle immobilization in this work.

**Figure 1:**
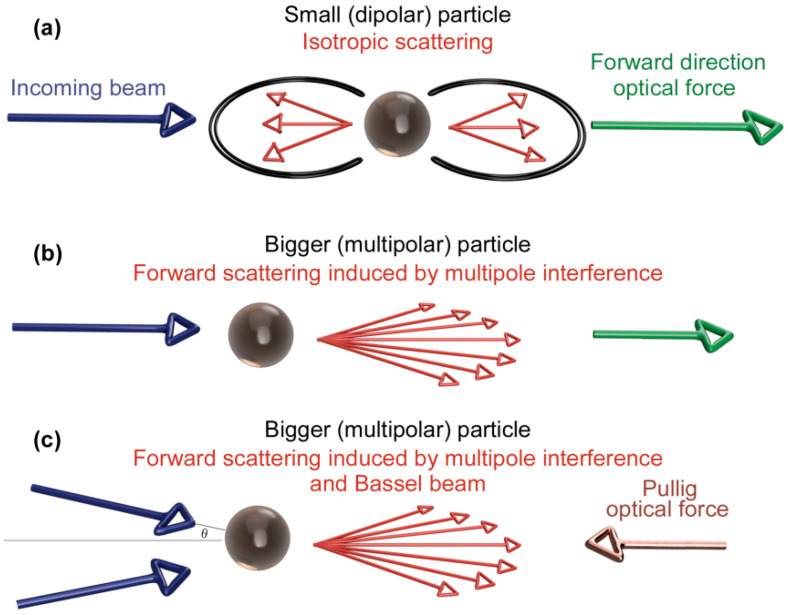
Scattering forces acting on rounded dipolar and multipolar metal nanoparticles. Isotropic scattering on small dipolar particles; arise of forward-directed optical force (**a**). Mutual interference between multipolar fields and particles in highly collimated beams; arise of reduced forward-directed optical force (**b**). Attenuation of radiation force with increasing angle between incoming beams; arise of optical pulling force (**c**).

Typically, depending on the size of the spherical metal nanoparticle, three scenarios of acting forces resulting from scattering on dipolar and multipolar particles may occur (see Figure 1), while only two of them lead to an ordered movement of particles due to the domination of the so-called forward scattering force [23]. This effect is multiplied once the incoming light is polarized. Different cases should be considered: (i) In the case of small silver nanoparticles, with diameter < 60 nm [27], dipolar plasmon resonance dominates, which leads to both absorption and isotropic scattering of incoming electromagnetic radiation. Isotropic scattering of light gives a net force on the particle in the forward direction, i.e., in the direction of the incoming beam, due to above-mentioned conservation of total momentum (Figure 1a). (ii) Interference between multipolar fields in multipolar particles which can strongly orient the scattering in the forward direction in the presence of a highly collimated beam (a plane wave). For spherical silver nanoparticles, multipolar localized surface plasmon resonance (LSPR) takes place at particles with diameter above 60 nm [27]. Owing to momentum conservation [23,25], the total forward force is reduced compared to dipolar interaction, however it still remains dominant, giving the particles the same oriented momentum as in (i) the direction of radiation propagation (Figure 1b). (iii) In the case of non-collimated light (typically in highly focused beams), the radiation force is closely related to mutual angle *θ* between incoming beams. As the angle between the beams increases, the traditional radiation force (the force that is exerted upon any material due to the exchange of momentum between the object and the electromagnetic field) goes to zero with the cos *θ* (Figure 1c), whereas the contribution to the force coming from the strongly focused forward scattering remains finite [23]. Above a given angle, there is an optical pulling force that acts against the photon stream.

It is well known that optically active nano-objects exhibiting localized surface plasmon (LSP) are good candidates for thermal excitation by intense laser light operating at wavelengths close to LSP resonance maxima [28,29,30,31]. Pulsed *fs* lasers with pulse duration of hundreds of *fs* are of especially great potential, since the duration of the temperature rise is comparable to that of heat diffusion, which typically equilibrates the overall temperature within a nano-object in about 10^−12^ s [32]. This can induce large transient thermal gradients within nanoscale objects since heat generation and heat diffusion occur successively. Use of *fs* lasers may lead to thermal destruction of a nano-object due to overcoming their melting point [31]. On the other hand, temperature increases induced by *ns* laser may increase the temperature at LSP hotspots in the order of some tens of degrees, which is sufficiently high, when considering the usual “softening” temperature of semicrystalline polymers in the range of 70–130 °C.

## 3. Results and Discussion

In this work, the fundamental principle behind intense light-induced collective manipulation with silver nanoparticles is to be clarified. For this purpose, a wide range of commercially available polymeric foils were selected (PET, PEN, PA66, PI, PEEK), differing in chemical composition, physical parameters, and the ability to absorb the laser radiation at the used wavelength (248 nm). The use of this technique is demonstrated in the preparation of nanoparticle-enriched polymeric surfaces, which can be applied in the fight against biofilm formation on medical devices. Therefore, after thorough characterization of prepared nanoparticle–polymer composites, antimicrobial tests were performed on selected polymer using the drop plate technique.

### 3.1. Characterization of AgNPs Colloids

**Figure 2:**
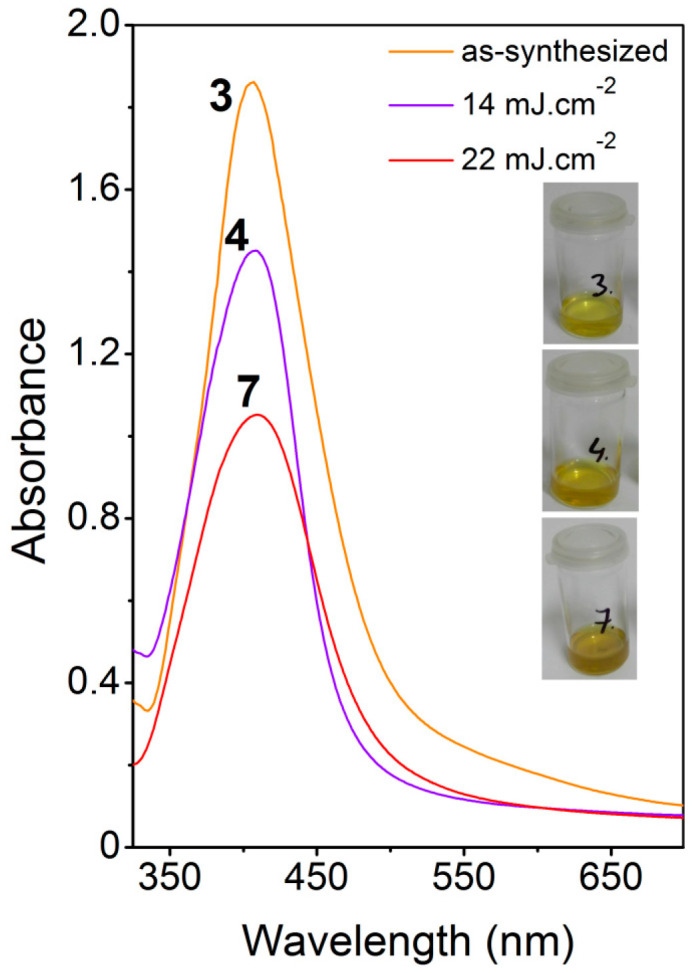
Absorption spectra of colloid solutions of electrochemically synthesized AgNPs in ultraviolet-visible region. As-synthesized solution (yellow curve-3), AgNPs colloid after laser irradiation at fluence 14 mJ cm^−2^ (purple curve-4), AgNPs colloid after laser irradiation at fluence 22 mJ cm^-2^ (red curve-4). Insets to the left show photographs of corresponding colloids.

As-synthesized AgNP colloid solution and solutions after the immobilization process were analyzed by UV–Vis spectroscopy (see Figure 2; insets show photographs of corresponding AgNP solutions). The as-synthesized solution (yellow curve) exhibited an intense monomodal peak with a maximum at 407 nm, characterized by rich yellow coloration, which corresponds to localized plasmon excited in the round shape silver nanopaticles with size around 20–25 nm [27]. In order to follow the changes taking place in the AgNP solution during the interaction with laser radiation, we also analyzed the solutions after immobilization process. A remarkable decrease in the peak intensity after the irradiation with laser fluence of 14 mJ cm^−2^ (purple curve) was mainly due to dilution of the as-synthesized solution to the desired Ag concentration of 30 mg/L. The peak maximum was shifted only to 408 nm, i.e., practically the same value as for as-synthesized AgNPs. This negligible shift indicates the fact that the absorbed laser irradiation did not affect the colloid itself. This can also be seen upon the solution coloration (see insets 3 and 4). A slightly different situation can be seen in the case of the AgNP solution after irradiation with higher laser fluence of 22 mJ cm^−2^ (red curve). The decrease of the peak intensity may be attributed to more pronounced incorporation of AgNPs at elevated fluence and resulting decrease of Ag concentration. One should also notice that the absorption peak was slightly broadened (increase in FWHM), suggesting a particle agglomeration. These changes were accompanied by shift of absorption maxima to 410 nm, which can also be seen in the change of the solution coloration (inset 7).

**Figure 3:**
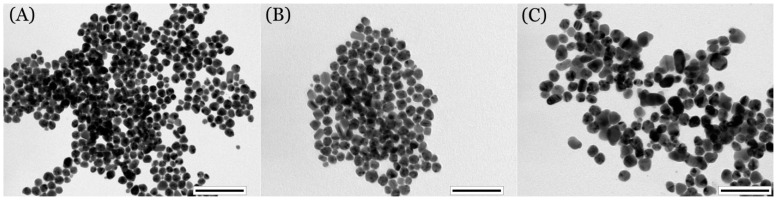
Images from transmission electron microscopy showing as-synthesized AgNPs (**A**) and AgNPs after laser irradiation at fluencies 14 mJ cm^−2^ (**B**), 22 mJ cm^−2^ (**C**). Scale bar is 100 nm.

To confirm the findings from the UV–Vis measurement, we performed a TEM analysis on the identical samples (see Figure 3). Figure 3A shows representative images of the as-synthesized AgNPs solution. It is obvious that the solution contained round-shaped AgNPs of narrow size distribution. The average size derived from TEM was 23.4 ± 4.2 nm, excluding size extremes with a frequency below 1%. The AgNP solution after irradiation with laser fluence of 14 mJ cm^−2^ exhibited similar characteristics, and neither the shape of the nanoparticles nor their size changed fundamentally (Figure 3B). A more significant change in the character of nanoparticles occurred after irradiation with 22 mJ cm^−2^. In accordance with UV–Vis findings, AgNPs tended to aggregate, they lost their characteristic round shape, and the size distribution broadened (Figure 3C). Similar changes in the shape and size distribution are typical for interactions of metal nanoparticles with high intensity light sources. Popov et al. [33] observed considerable changes in the size and morphology of isolated particles as well as their aggregation into multi-particle aggregates during the irradiation of AgNP colloids with an argon-ion laser. These changes are strongly sensitive to the type of stabilizing agent as well as to the NP/water ratio.

### 3.2. Laser Immobilization and Surface Characterization of Polymers

After laser irradiation, the polymer was removed from the immobilization solution, rinsed with distilled water, dried with a stream of nitrogen and placed into desiccator for at least 72 h, prior to each surface analysis (AFM, SEM, TEM).

**Figure 4:**
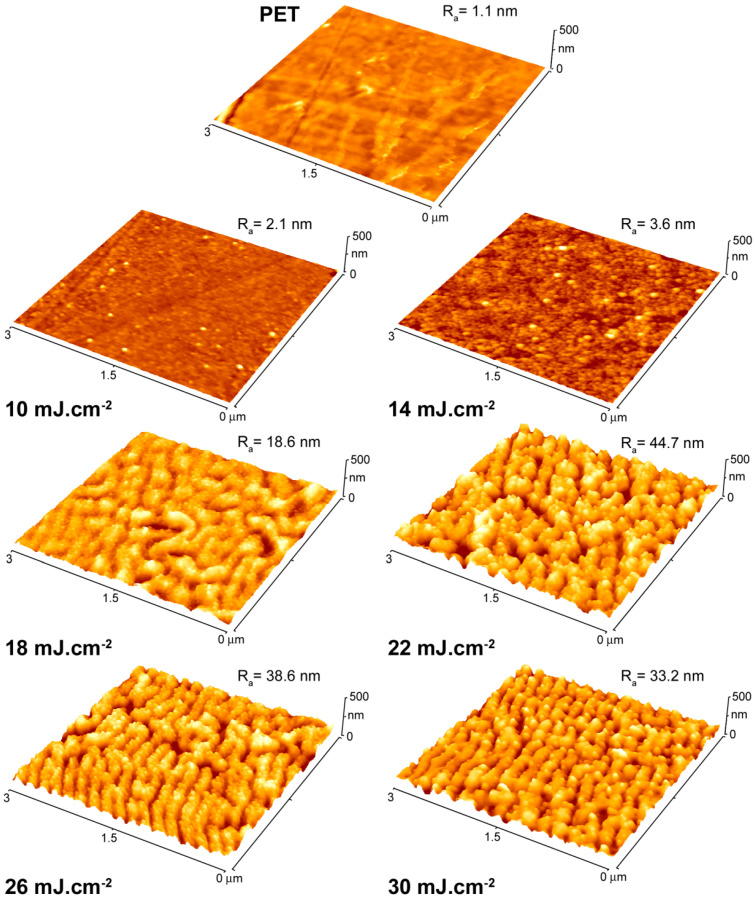
Images from atomic force microscopy showing surface morphology of pristine PET and AgNP-immobilized PET at laser fluence ranging from 10 to 30 mJ cm^−2^. *R*_a_ represents average surface roughness in nm.

We started our experiments using PET foil irradiated to laser fluencies from 10 to 30 mJ cm^−2^ (with a step of 4 mJ cm^−2^) in order to obtain detailed information on the development of surface morphology as a function of the specific fluence applied. For this procedure we took over the optimal parameters from our previous study, the results of which have been previously published [19]. The surface morphology of irradiated samples together with pristine PET as reference was recorded by AFM (see Figure 4) and corresponding values of surface roughness were calculated. It is obvious that with increasing fluence, the surface roughness increased and reached its maximum at the fluence of 22 mJ cm^−2^, then surface roughness slightly decreased. This phenomenon was accompanied by an increase of another surface characteristic, i.e., effective surface area, which represents the real surface area of a scanned square. Effective surface area for PET showed the similar maximum of 10.1 μm^2^ for the laser fluence of 22 mJ cm^−2^. In the view of possible applications of such material in e.g., bacteria inhibition, the higher surface area is expected to lead to more effective inhibition action. Therefore, the laser fluence of 22 mJ cm^−2^ was chosen as optimal for corrugated structures. On the other hand, the highest possible fluence (14 mJ cm^−2^) with no significant impact on the planar nature of the polymer was selected as a parallel one for further experiments.

The experiments on PET make it possible to narrow the range of laser fluencies, with two subsequently applied to other polymers i.e., 14 and 22 mJ cm^−2^, preserving either smooth polymer surface morphology and leading to pronounced surface corrugation. The development of specific surface corrugation of polymers under higher fluencies is due to their light absorption owing to the presence of conjugated double bones in the polymer chain [34], more specifically due to the presence of benzene nuclei. Thus, apart from PET, changes in surface morphology under higher fluencies can also be expected in the case of PEN, PEEK, and PI.

**Figure 5:**
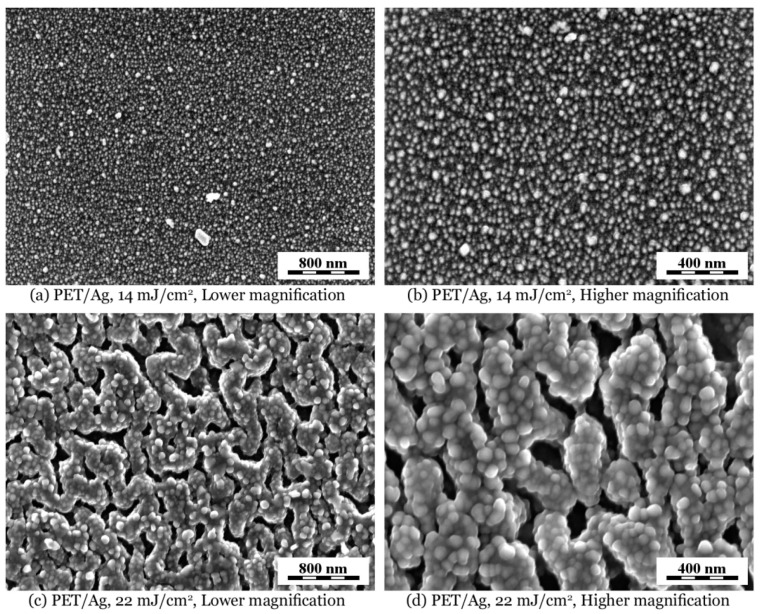
FEGSEM micrographs showing surface morphology of PET immobilized with AgNPs at laser fluencies of 14 and 22 mJ cm^−2^: (**a**,**b**) smooth PET and (**c**,**d**) corrugated PET surface. Micrographs were recorded with in-beam SE detector at accelerating voltage 3 kV.

The surface morphology of AgNP-decorated polymers was observed by FEGSEM microscopy combined with EDX microanalysis. Figure 5 shows smooth and corrugated PET films with immobilized Ag nanoparticles under fluencies 14 and 22 mJ cm^−2^. Lower magnification micrographs (Figure 5a,c) illustrate that the morphology was quite uniform, while higher magnification micrographs (Figure 5b,d) display incorporated AgNPs at better resolution. It is obvious that the irradiation with higher fluence resulted in a rough and rugged polymer surface with bigger AgNPs, which is in good accordance with the TEM analysis of the immobilization solution of AgNPs after laser irradiation (see Figure 3C). It is also seen that regardless of the nature of the polymer surface after nanoparticle immobilization (smooth or rough), the AgNPs are always located at the very surface area of the polymer.

**Figure 6:**
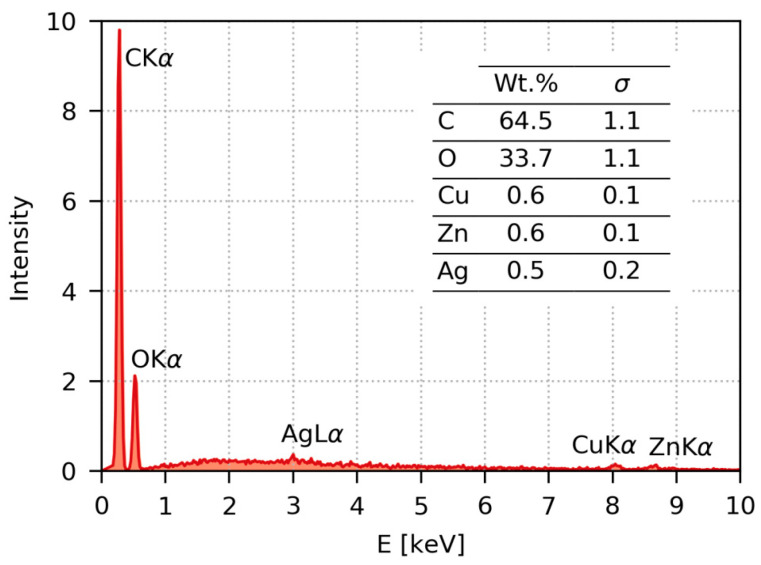
Typical EDX (energy-dispersive analysis of X-rays) spectrum recorded from the surface of PET immobilized at laser fluence of 14 mJ cm^−2^. The inset table shows results of semi-quantitative standardless EDX analysis.

Figure 6 shows the typical EDX spectrum recorded from the surface of the AgNP/PET thin film. The spectrum is dominated by peaks from the polymer (C and O). The peak corresponding to deposited nanoparticles (Ag) appears just slightly above the noise level, confirming that the nanoparticle layer was quite thin. It should be noted that the penetration depth of primary electrons accelerated at 30 kV in polymer samples is around 20 μm, while the AgNP layer is just a few nm thick. The two remaining small peaks at higher energies (Cu and Zn) correspond to the weak signal from the brass support on which the polymer film was fixed.

**Figure 7:**
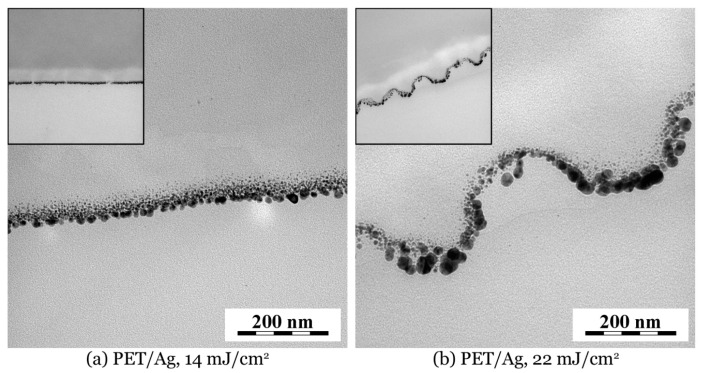
TEM micrographs of cross sections of AgNP-immobilized PET: (**a**) smooth PET and (**b**) corrugated PET surface. In both micrographs and insets, the polymer phase is on top and embedding epoxy resin is at the bottom of the image. Real width of both insets is 1.68 μm.

**Figure 8:**
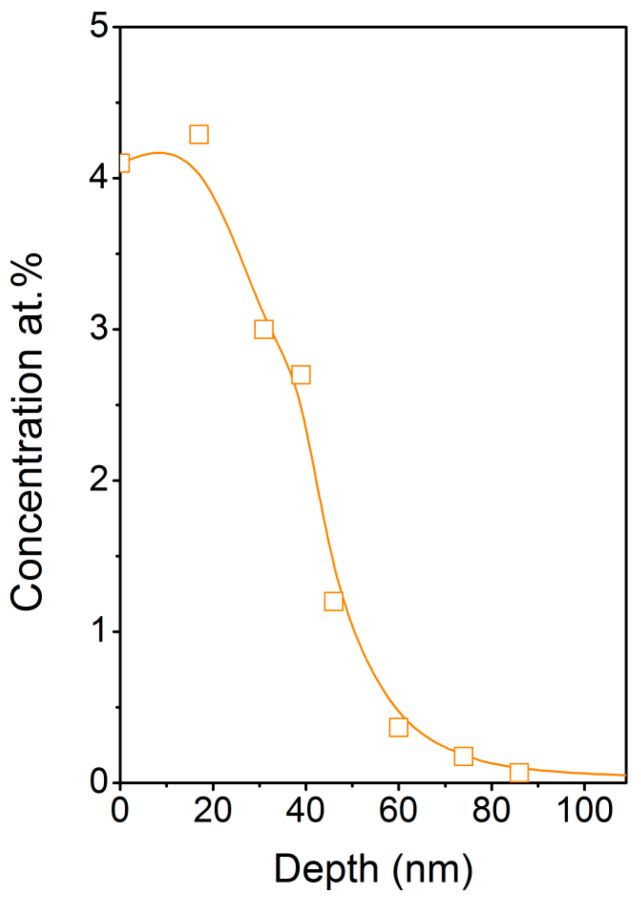
Concentration profile of Ag in AgNP-immobilized PET (14 mJ cm^−2^) measured by Rutherford backscattering spectrometry (RBS).

In order to clarify anchoring method and penetration depth of AgNPs in PET we performed TEM analysis of a AgNPs/PET cross section. TEM micrographs displayed in Figure 7 show the penetration of AgNPs into the PET films. In agreement with SEM micrographs, the TEM confirmed that on the PET irradiated with fluence 22 mJ cm^−2^, slightly enlarged AgNPs are located on non-flat, rugged surface sites. Moreover, TEM micrographs show that the biggest AgNPs were localized closer to the surface, while smaller ones tended to penetrate deeper, as could be expected. Average AgNP penetration depth was about 70–80 nm, while the smaller particles penetrated deeper and their size increased towards the polymer surface. In addition to the TEM cross section analysis we also determined RBS profiling of Ag in PET (Figure 8). Since surface microstructure may significantly distort a measured concentration profile [35], we performed RBS analysis on a PET sample immobilized with 14 mJ cm^-2^. One can clearly see that maximal particle concentration was in the surface area of the polymer and decreased monotonically towards the polymer bulk. While AgNP penetration depth was about 70–80 nm, practically no silver was detectable at 90 nm depth, which is in good agreement with the TEM analysis.

**Figure 9:**
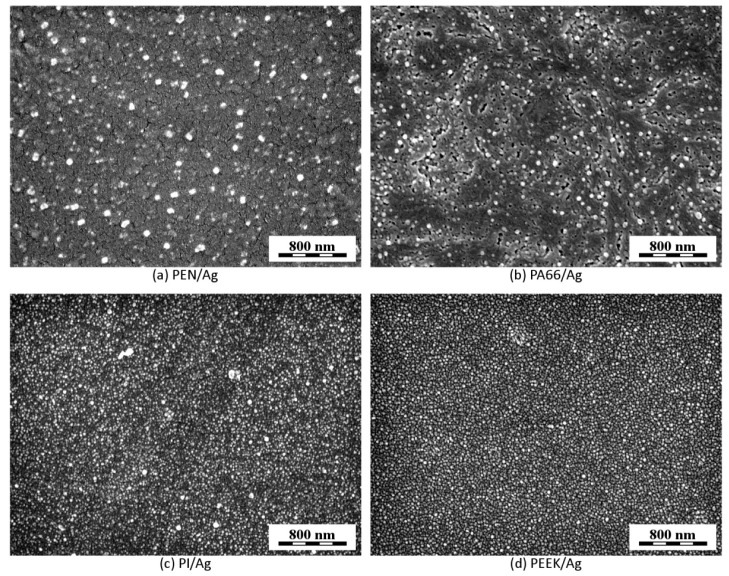
FEGSEM micrographs of surface morphology of polymers immobilized with AgNPs (laser fluence 14 mJ cm^−2^): PEN (**a**), PA66 (**b**), PI (**c**), and PEEK (**d**). All micrographs were recorded with in-beam SE detector at accelerating voltage 3 kV.

Figure 9 displays surface morphology of AgNP-decorated polymer films for PEN (Figure 9a), PA66 (Figure 9b), PI (Figure 9c), and PEEK (Figure 9d), using immobilization fluence of 14 mJ cm^−2^. Surface morphology of all investigated polymers was quite similar, as polymers preserved the smooth character of the surface, but they were quite different in the AgNP size and distribution over the surface. PEN and PA66 polymers (Figure 9a,b) contained a mixture of bigger and smaller AgNPs on their surfaces, probably due to more pronounced size-dependent segregation of AgNPs during their penetration into polymers, whereas PI and PEEK polymers (Figure 9c,d) exhibited much narrower AgNP size distribution, similar to that observed for PET decorated under the same conditions (compare Figure 5a,b with Figure 9c,d). In line with our expectations, PEN, PEEK, and PI exhibited corrugated morphology at higher immobilization fluence, however these data are not shown, since such results are at the moment hardly reproducible and applied laser fluence needs to be optimized in the sense of these polymers in further experiments.

The information from image analyses (SEM, TEM) can be supplemented with the data on elemental composition of the surface determined by the XPS method. Atomic concentrations of elements are summarized in Table 1. Besides elements typical for pristine polymers (C, O, and N), the changes in silver concentration depending on the treatment conditions are particularly interesting. With no exception, the higher the laser fluence, the higher the concentration of Ag on the polymer surface. This finding corresponds to the assumption that higher fluence leads to higher particle temperature which in turn results in greater tendency of particles to aggregate. Concentration of C, O, and N in pristine polymers corresponds well with theoretical element concentrations given by polymer stoichiometry. It is apparent that once the silver is detected, it primarily occurs at the expense of carbon. This is probably due to the simultaneous enrichment of the polymer surface with oxygen-containing functional groups. This finding is in contradiction to our previously published data on underwater laser treatment of PET [34], suggesting the role of silver on this phenomenon. The physical principle behind the particle immobilization mechanism itself will be discussed in detail later.

As discussed in the introduction and proved by SEM and TEM analysis, the nature of the AgNP/polymer interface (localization of silver particles a few tens of nm beneath the surface) predestines these materials for use in medical applications as promising inhibitors of bacterial infections. To reveal the bactericidal potency of the developed materials, we performed standardized antibacterial tests using the drop plate method on PET, whose morphology as well as AgNP distribution were analyzed in detail. We examined PET foils treated at 14 and 22 mJ cm^−2^ fluencies, exhibiting both smooth and corrugated surface morphology enriched with AgNPs.

### 3.3. Antibacterial Potency of AgNPs Decorated PET

The antibacterial effects of PET, PET underwater laser-treated (fluence of 14 mJ cm^-2^) with specific surface structure but without immobilized AgNPs (PET/H_2_O_14), and PET with AgNPs prepared under immobilization fluencies of 14 (PET/AgNPs_14) and 22 mJ cm^−2^ (PET/AgNPs_22), were evaluated by the drop plate method against *Escherichia coli* and *Staphylococcus epidermidis*, common representatives of Gram-negative and Gram-positive bacteria, respectively. The advantages of this method are namely its simplicity and execution speed [36,37,38,39,40,41].

**Figure 10:**
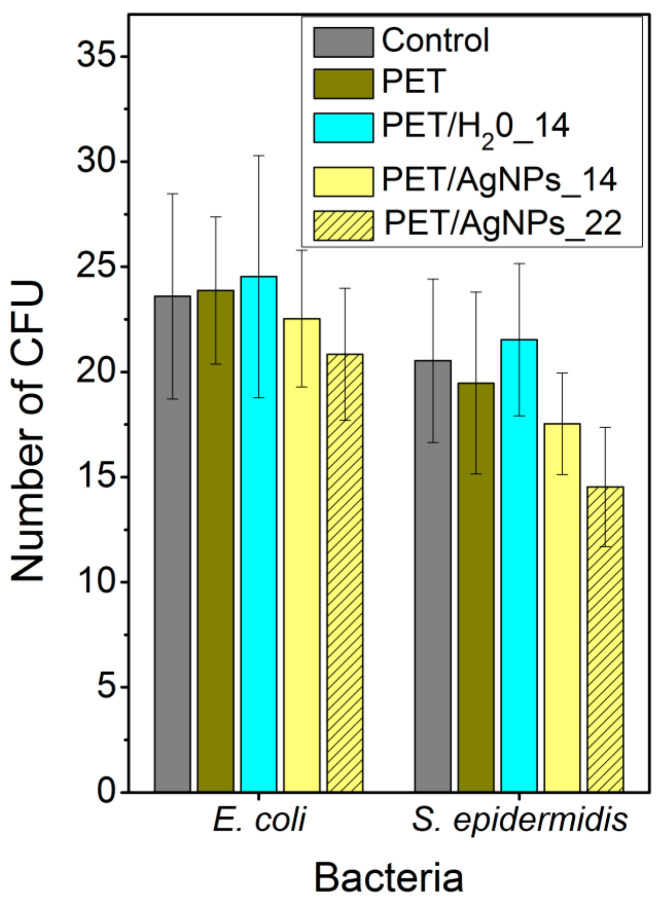
Antibacterial tests performed on pristine PET (PET), underwater laser-treated PET without embedded AgNPs at laser fluence 14 mJ cm^−2^ (PET/H_2_O_14), and AgNP-immobilized PET at fluencies of 14 and 22 mJ cm^−2^. Tests were evaluated on *E. coli* and *S. epidermidis* and antibacterial efficacy was measured by number of colony-forming units (CFU). Control bar represents CFU of bacteria in pure physiological solution.

The results of the antibacterial tests are summarized in Figure 10. Surprisingly, none of tested samples exhibited significant antibacterial effects against *E.coli* or *S. epidermidis*. It is obvious that the presence of the typical surface structure resulting from underwater laser treatment itself [34] did not evoke any antibacterial effect. Only in the case of PET/AgNPs_22 was the number of CFU slightly smaller than the control (bacteria cultivated in pure physiological solution), but nevertheless it was still within the error range. Regarding this fact, that the concentration of Ag ions detected in leachate (Table 2) was by three orders of magnitude less than the minimum inhibitory concentrations (MIC), one may attribute the observed antibacterial effect to the synergy between the rough surface and the presence of the antibacterial component itself. This conclusion can be supported by the literature data [42], reporting the MIC of silver, effective against single-cell organisms, from 0.1 to 20 mg/L. More specifically, the MIC for *E. coli* typically ranges from 3.50 mg/L (Ag^+^) to 13.02 mg/L (AgNPs), and for *S. epidermidis* about 6.25 mg/L (AgNPs, depending on their size and shape). The fact that the tested samples exhibited almost no antibacterial effects corresponds with the general stability of the AgNP attachment to polymer support and low formation ability of Ag ions, which are released into the surrounding media (see Table 2). These properties may implicate potential uses of developed materials in clinical practice [43,44,45], where the stability of the AgNP/polymer interface may be advantageous in prevention of biofilm formation [46,47] with long-term stability. Therefore, our further experiments address the ability of newly developed surfaces to prevent and inhibit biofilm formation.

### 3.4. Mechanism of AgNPs Incorporation into Polymers

#### 3.4.1. Radiation Pressure and Absorption Cross Section

The specific experimental set up (small metal spherical nanoparticles, plane-wave source) perfectly coincides with the assumptions of the classical Mie scattering theory [48]. This analytical approach allows us to calculate both an absorption cross section and radiation pressure [49], both needed to describe our experiment. This method is well established and widely available, providing the most complete description of the small particle interaction with light.

**Figure 11:**
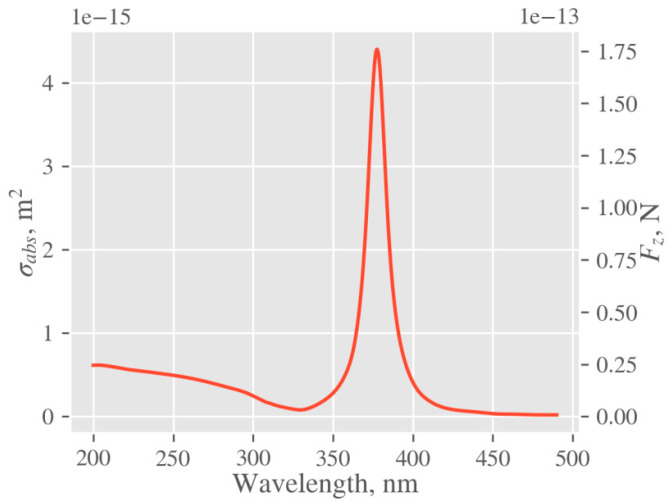
Absorption cross section (*σ*_abs_) and optical force (*F*_z_) of the modeled silver nanoparticle calculated in *Z*-direction according to Mie theory.

Forces and absorption properties of silver nanoparticles (NPs) were calculated in the Mie theory formalism, using PyMieScatt implementation [50]. Optical properties of silver were taken from [51]. The geometrical description comprises a silver sphere of diameter ~23 nm (mode of the distribution) submerged into water. The calculated plot of absorption cross section and radiation force is shown in Figure 11. Since the excimer laser used in our experiments has a pulse duration of 25 ns, spectral broadening due to energy–time uncertainty is negligible, and thus the values of the force and absorption cross sections corresponding to the wavelength of 248 nm were used. Excited optical force at 248 nm is about 25 pN, which is seven times lower than its maximum at 380 nm. However, with respect to NP size, it is still quite enough to induce collective motion of nanoparticles towards the polymer surface [52,53]. Moreover, the fact that we conducted our experiments at 248 nm, where the absorption cross section is considerably lower than the maximal one, enables us to tailor the surface morphology of polymers by light absorption. The reason lies in the favorably low values of the absorption cross section and the rare interaction of laser light (absorption) with AgNPs. Thus, the residual beam energy can act on the polymer surface giving arise to a specific corrugation at higher fluencies, if only the polymer absorbs.

#### 3.4.2. Local Temperature of Ag Nanoparticles

**Figure 12:**
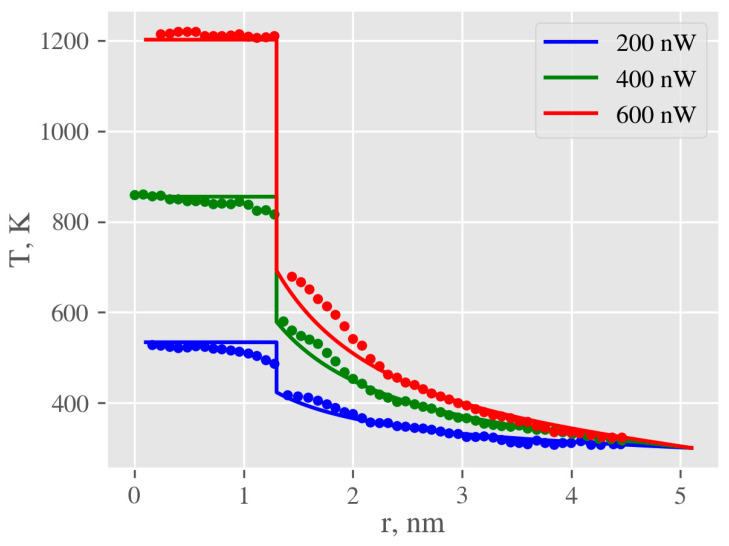
Comparison of temperature profiles in the AuNP-octane system obtained by continuum model (solid lines) with those calculated by molecular dynamics (dots), using finite element method (FEM). Calculations were done for total absorbed energies 200, 400, and 600 nW. Data for calculation were taken from [55].

The problem of heat transfer from AgNPs is determined by the thermal properties of the surrounding medium (which are readily available) and properties of the interface, characterized by the Kapitza conductance (*G*), which defines the relationship between temperature gradient on the interface and heat flow through it. Unfortunately, it is impossible to measure temperature directly at the nanoscale, thus molecular dynamics (MD) simulations are usually used. The problem of heat transfer between AgNPs and water was intensively studied by Rajabpour et al. [54], who calculated values of interfacial thermal conductance for that system. Other important data, temperature profiles for nanoparticles submerged in different media, were determined by MD simulations in [55]. To estimate the meaningfulness of the continuum model, used for all further calculations, temperature profiles for the gold-octane system were calculated using finite element method (FEM) and compared with the data provided in [55] (Figure 12). Macroscopic constants of gold and octane were used and only Kapitza conductance values (*G*) were taken from the publication [54]. The comparison of these two methods suggests that although the continuum model is unable to capture small local increases in temperature in the vicinity of the NP (arising from enhanced density of the adsorbed liquid layer) it is precise enough for further calculations, providing an accurate description for the temperature of the NP core and surrounding liquid for distances above 2 nm.

We were unable to find any MD simulations of the heat transfer from metal NPs to polymers. The closest investigated problem may be heat transfer from silicon to polyethylene, studied by Hu et al. [56], who reported the value of interfacial conductance of about 20 MW m^−2^·K^−1^. At the same time, there are several reports of experimental measurements of the Kapitza conductance of the polymer-crystal interface, also shown in Table 3. Despite great differences between these values, they enable one to estimate the order of magnitude for the interfacial conductance.

**Figure 13:**
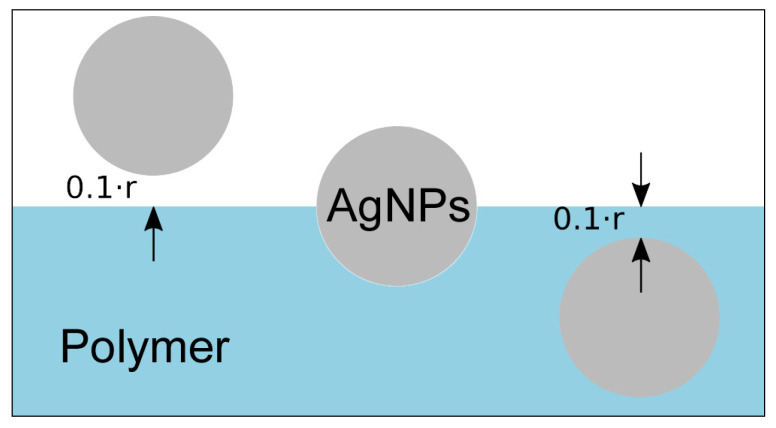
Geometry of three limit positions of AgNPs relative to the polymer surface (from left to right): nanoparticle in the water in the vicinity of polymer, half-submerged into polymer surface, and completely submerged.

**Figure 14:**
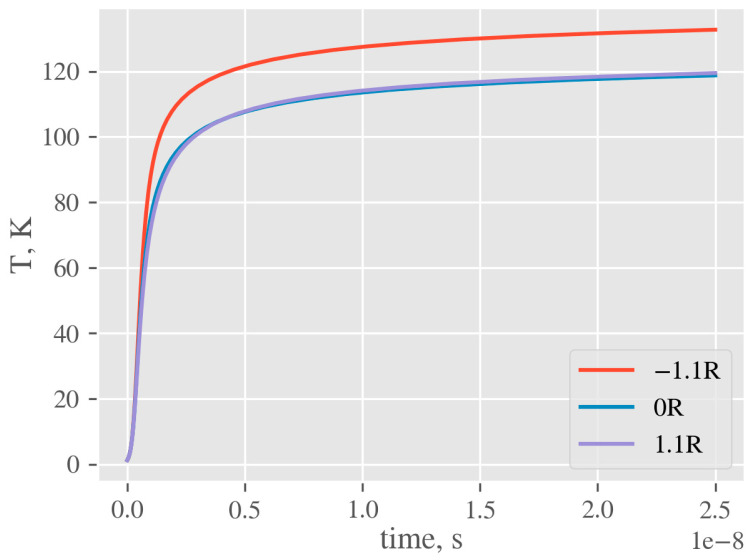
Temperature evolution of AgNP core for different immersion depths (PET, G = 50 MW m^−2^ K^−1^).

**Figure 15:**
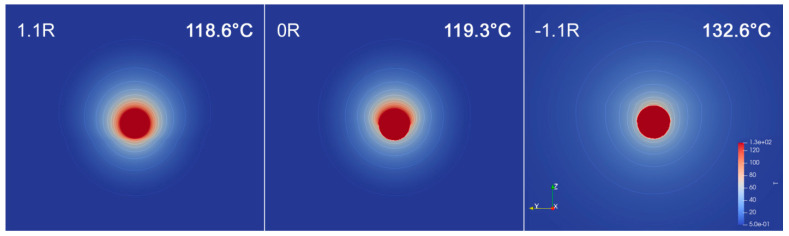
Temperature cross section of AgNPs at the end of laser pulse with respect to their immersion depth in PET (1.1r, 0r, −1.1r, according to Figure 14). Insets in right upper corner refer to maximal temperature in the nanoparticle core.

To estimate temperature elevation in the vicinity of NP, the heat equation was solved using the FEM, implemented in FlexPDE [49]. The laser pulse shape was approximated by a rectangle. The geometry used in simulation includes NP of diameter 23 nm in the vicinity of polymer (surface-to-polymer center distance 1.1*r*), half-submerged in polymer (0*r*), and completely submerged in the polymer (−1.1*r*, see Figure 13). Heat is generated in the whole volume of NP with the rate equal to the product of laser intensity and NP cross section. All material parameters are assumed to be temperature-independent. The simulation is started from the equilibrium state at the reference temperature (equal to 0) and continued until the end of the laser pulse. The temperature on the outside of the computational cell was fixed at 0 and corresponding interfacial conditions were applied. Kapitza conductance from [54] was used to define contact conductance of nanoparticle–water interface. Since the literature data on the conductance at the nanoparticle–polymer interface vary significantly, the calculations were simulated for values of 10, 25, 50, 75, 100 MW m^−2^·K^−1^ (the same value is assumed for all simulated polymers). To illustrate how the specific value of Kapitza conductance affects resulting nanoparticle temperature at the end of laser pulse, we plotted calculated temperature *T* as a function of *G* for all examined polymers and AgNPs positioned at 0*r* (Figure 14). An example of temperature evolution of the AgNP core within a single laser pulse for different immersion depths in PET (according to Figure 13) and Kapitza conductance *G* = 50 MW m^−2^ K^−1^ is shown in Figure 15.

#### 3.4.3. Increase of Temperature of AgNP Colloid on Macroscopic Level

**Figure 16:**
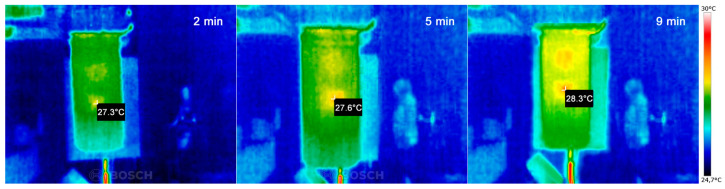
Images from thermo camera showing heating of AgNP colloid solution during irradiation process after 2, 6, and 9 min.

The temperature increase within the volume of cuvette containing an AgNP colloid due to heat transfer from nanoparticles to the liquid environment was measured by a thermo camera. Those data are presented in Figure 16. One can see that within the interaction area, the local temperature increased monotonically with irradiation time. It is obvious that after the 9 min of exposition the temperature increase in the hotspot was approximately 1 °C. At the same time, considerable heat diffusion out from the interaction area was detected, causing a temperature increase of the liquid also outside the interaction volume defined by aperture.

To confirm the formal correctness of the calculations, we compared experimental data with the calculated temperature increase on macroscopic level taking into account the total absorbed energy supplied by the light source. Macroscopic heating was calculated by balancing average heat, provided by laser *P_laser_* and thermal losses *P_loss_*, i.e., perfect equilibration of system between pulses was assumed. The accumulation component is the heating of the solution of mass *m*_w_, having thermal capacity *c_p,w_* and silica cuvette of mass *m*_c_ and capacity *c_p,c_*. Provided that thermal losses consist only of convection, described as *P_loss_* = *K_conv_ A* Δ*T*, we get the differential equation

*d* Δ*T* = *dτ* (*P_laser_* − *P_loss_*)/(*c_p,w_m_w_* + *c_p,c_m_c_*) = *dτ* (*P_laser_* − *K_conv_A* Δ*T*)/(*c_p,w_m_w_* + *c_p,c_m_c_*) (1)

Solution of (1) is given by

**Figure 17:**
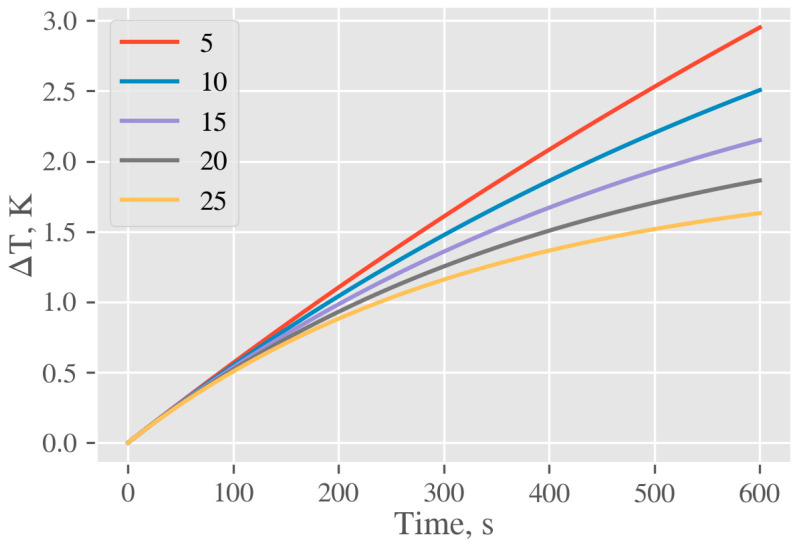
Calculated temperature profiles of AgNPs colloid solution in experimental cuvette due to heat transfer from NPs for typical values of the heat transfer coefficient *K*_conv_ (5, 10, 15, 20, 25) W m^−2^ K^−1^.

Δ*T*(*τ*) = *α*/*β*(1 − e^−*βτ*^) (2)

where *α* = *P_laser_*/(*c_p_,_w_m_w_* + *c_p,c_m_c_*) and *β* = *K_conv_ A* Δ*T*/(*c_p,w_m_w_* + *c_p,c_m_c_*). The only parameter in Equation (2) which cannot be exactly determined is a convective heat transfer coefficient *K_conv_*. According to [60] it typically lies in the range from 2.5 to 25 W m^−2^ K^−1^, thus calculations were done for a set of different values from this interval. Obtained curves are plotted in Figure 17. Calculated data confirm a good agreement between predicted and experimental macroscopic increases of temperature measured by the thermo camera. The small discrepancy between these two values may be due to (i) inability to determine the coefficient *K_conv_* exactly and/or (ii) impossibility to identify all heat loss in cuvette open system and/or (iii) our premise that the energy of the beam is fully absorbed. However, the reasonable agreement of both values points to the correct assumptions and the calculated temperature rise of AgNPs due to the conversion of light radiation to heat can be considered a correct one.

Therefore, upon the simulations and calculation results presented above, we may formulate the mechanism of incorporation of nanoparticles in the surfaces of the polymer layer as follows. Due to the conversion of light energy into heat, the particles increase their temperature up to 120–140 °C and simultaneously, due to the conservation of the momentum of the scattered radiation, they are collectively pushed along the light propagation against the polymer surface. Although the pushing force as well as the absorbed heat increase monotonically with nanoparticle size, the mechanical resistance of the polymer, preventing bigger particles from penetrating deeper into its volume, grows quicker than the force itself. This leads to the observed sorting of NPs in the volume of the polymer according to their size, i.e., the deepest incorporated particles are the smallest ones and their average size increases towards the polymer surface.

## 4. Experimental

### 4.1. Materials, Apparatus and Procedures

AgNPs were prepared electrochemically under the slightly modified procedure referred to in our previous work [19]. In this case, more uniform and stable round-shape AgNPs with an average size of about 23 nm were produced. Two silver electrodes (silver bars, dimensions of 40 × 10 × 1 mm^3^, purity 99.99%, supplied by Safina a.s., Czech Republic) were immersed in sodium citrate electrolyte (0.4 mM, volume 150 mL, supplied by Sigma-Aldrich Co., St Louis, MO, USA) and powered by DC power supply (voltage 15 V, current 150 mA). DC voltage was applied to the silver bars immersed in electrolyte for 0.5 h under vigorous magnetic stirring at room temperature. Afterwards, the silver electrodes were carefully removed to avoid the dispersion of silver agglomerates from the cathode, caused by imperfect transport of formed NPs into the liquid volume. The beaker containing the solution was kept for 24 h in darkness to complete AgNP formation. The AgNP solution was then decanted and filtered using nylon filter syringe attachments (Rotilabo, Carl Roth GmbH, Karlsruhe, Germany, pore size 0.1 µm) to remove macroscopic impurities. Finally, the concentration of Ag in the prepared colloid NP solution was determined by atomic absorption spectroscopy. Under these conditions, the typical Ag concentration in the AgNP colloids was around 40 mg/L. As-synthesized AgNP solutions were adjusted to working concentration (30 mg/L) by dilution with buffer solution (water solution of sodium citrate, 0.4 mM).

**Figure 18:**
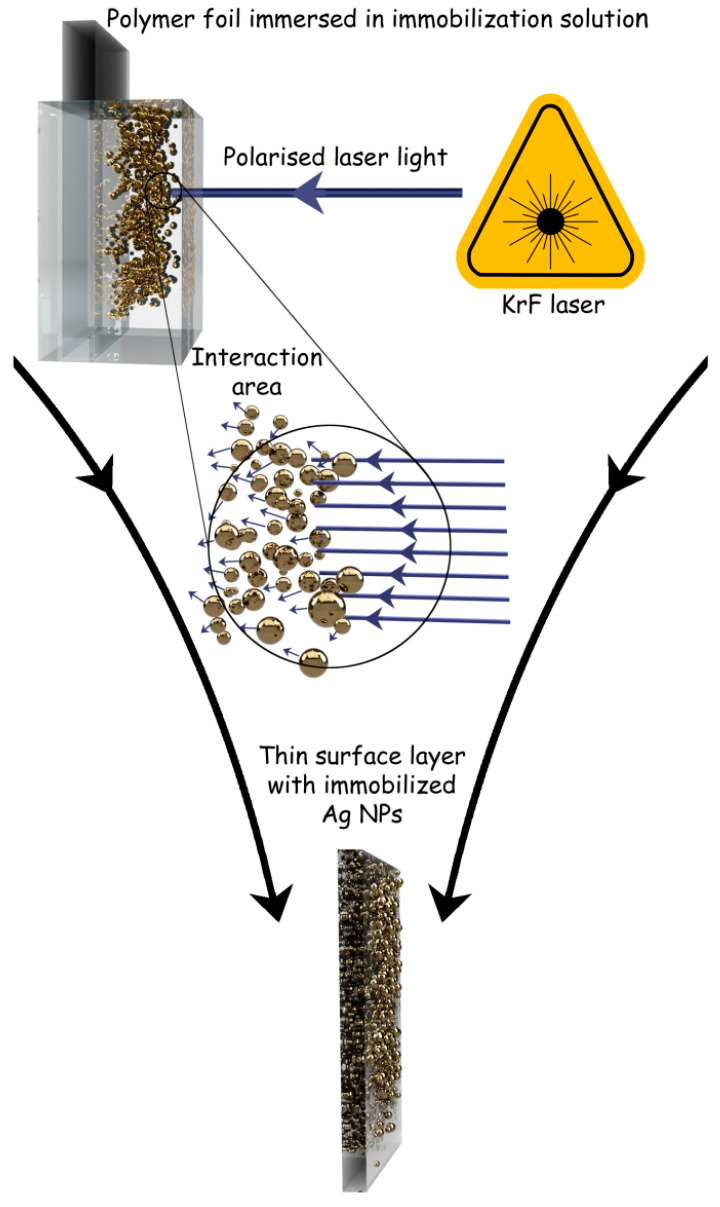
Scheme of the process of immobilization of silver nanoparticles (AgNPs) on polymers by the action of laser beam, resulting in a thin surface layer of polymer enriched with AgNPs.

Immobilization of silver NPs was carried out using a KrF excimer laser (COMPex Pro 50F, Coherent, Inc., Silicon Valley, CA USA, wavelength 248 nm, pulse duration 25 ns, and repetition rate 10 Hz, laser fluencies from 10 to 30 mJ cm^−2^). A strip of polymer foil (Goodfellow Ltd., UK, thickness 50 μm, dimensions 30 × 8 mm) was cut and placed vertically into high precision spectroscopic cells (HellmaAnalitics GmbH, Mullheim, Germany, type No. 100-QS, light path 10 mm) so that the polymer strip was centered in the middle of the cuvette. Afterwards, 3.5 mL of colloidal AgNP solution was added using an automatic pipette. The laser light was linearly polarized with a UV-grade fused silica prism (model PBSO-248-100). Irradiation was performed perpendicularly to the PET (polyethylene terephthalate) surface, using an aperture with an area of 5 × 10 mm^2^. The scheme of polymer processing with laser light is shown in Figure 18. Treated polymers in this work were polyethylene terephthalate (PET), polyethylene naphthalate (PEN), polyamide 66 (PA), polyimide (PI), and polyetheretherketone (PEEK).

### 4.2. Materials, Apparatus and Procedures

The concentration of Ag in prepared colloid NP solution was determined by atomic absorption spectroscopy (AAS) on a VarianAA880 device (Varian Inc., Palo Alto, CA, USA) using a flame atomizer at 242.8 nm wavelength. The typical uncertainty of concentration determined by this method was less than 3%.

Ultraviolet–visible spectroscopy (UV–Vis) was used to study the optical properties of colloidal dispersions of AgNPs. Absorption spectra were recorded on a Lambda 25 spectrophotometer (PerkinElmer Inc., Waltham, MA, USA) in spectral range 300–700 nm with a 1 nm data step, scan speed of 240 nm/min. Measurements were accomplished in a polystyrene cuvette with 1 cm light path.

Silver NPs were analyzed by transmission electron microscopy (TEM) on JEOL JEM-1010 (JEOL Ltd., Akishima, Japan) operated at 400 kV. A drop of colloidal solution was placed on a copper grid coated with a thin amorphous carbon film on a filter paper. The excess of solvent was removed. Samples were air-dried and kept under vacuum in a desiccator before placing them on a specimen holder. Particle size was measured from the TEM micrographs and calculated by taking into account at least 500 particles.

Direct measurement of a temperature increase in the cuvette volume due to light-to-heat conversion in AgNPs during the process of laser irradiation was accomplished by thermal camera BOSCH GTC 400 (Robert Bosch GmbH, Stuttgart, Germany), operating in autocalibration mode of temperature sensor. In all experiments, the thermal camera was placed in the axis of the laser beam behind the cuvette.

Surface morphology of the samples was visualized by a high-resolution FEGSEM microscope (MAIA3, TESCAN, Brno, Czech Republic). The microscope was equipped with detectors of secondary electrons (SE), backscattered electrons (BSE), and detectors for energy-dispersive analysis of X-rays (EDX; detector X-MaxN 20; Oxford Instruments, UK). The samples for FEGSEM microscopy were small pieces of polymer film, attached with a double adhesive carbon tape (Cristine Groepl, Austria) to a brass stub and covered with a thin carbon layer in a vacuum evaporation device (JEE-4C; JEOL, Akishima, Japan). The samples were observed in the FEGSEM microscope using UH Resolution mode at accelerating voltage 3 kV and in-beam SE detector. Selected samples were also characterized by elemental microanalysis, when the accelerating voltage was increased to 30 kV and EDX spectra from the surface area were recorded.

Penetration of AgNPs into polymer surface was visualized by a TEM microscope (Tecnai G2 Spirit Twin, FEI, Brno, Czech Republic). Thin polymer films decorated with AgNPs were embedded in a single component epoxy resin (Durcupan; Sigma Aldrich, St Louis, MO, USA) and ultra-thin sections perpendicular to the surface with AgNPs were cut with an ultramicrotome (Austria) at room temperature. The thin sections were collected on carbon-coated copper TEM grids (Christine Groepl, Tulln, Austria) and observed in the TEM microscope using bright field imaging at accelerating voltage of 120 kV.

Surface morphology and roughness was also measured by atomic force microscope (AFM) on DimensionIcon (Bruker Corp., Billerica, MA, USA) in ScanAsyst-Air mode. A silicon nitride tip for ScanAsyst mode (Bruker Corp., Billerica, MA, USA) was used, operating near its resonant frequency of 70 kHz (spring constant 0.4 Nm^−1^). The scans were acquired at the line scanning rate of 0.5 Hz. Surface roughness, characterized by the mean roughness value (*R*_a_), represents the arithmetic average of the deviation from the center plane of a sample.

The Rutherford backscattering spectrometry (RBS) spectra were measured on a 3 MV Tandetron MC 4130 accelerator using 2.0 MeV 4He^+^ ions at a laboratory scattering angle of 170° in the Cornell geometry. The Ultra-Ortec PIPS detector solid angle was 2.612 mSr, the spectrometer energy resolution for 2.0 MeV 4He^+^ ions was defined at full width at half maximum (FWHM) = 12 keV and the beam spot area on the sample was 1x1 mm^2^. The typical beam current was 5 nA.

Atomic concentrations of elements on the surface of samples were determined by X-ray photoelectron spectroscopy (XPS). The measurement was carried out on an Omicron Nanotechnology ESCAProbe P spectrometer (Omicron Nanotechnology GmbH, Taunusstein, Germany) at pressure of 2 × 10^−8^ Pa. The atomic concentrations of silver Ag3d, carbon C1s, oxygen O1s, and nitrogen N2p in pristine and laser-irradiated/AgNP-immersed samples were analyzed. The X-ray source was monochromated at 1486.7 eV with the step size of 0.05 eV. Photoelectrons were collected under the take-off angle 90°. Scan size was 2 × 3 mm^2^ and spectra evaluation was carried out by CasaXPS software. The uncertainty of the measurement was less than 3%.

Inductively coupled plasma with mass spectroscopy detector (ICP-MS) was used to determine the bond strength of immobilized nanoparticles on the PET surface. The samples were immersed in 10 mL of distilled water and sonicated at 24 °C for 24 h. The trace element analysis of Ag leachates was conducted using an Agilent 8800 triple-quadrupole spectrometer (Agilent Technologies, Santa Clara, CA, USA) connected to an autosampler. Sample nebulization was performed using a MicroMist device equipped with a peristaltic pump. To minimize the interference of an analyte with ArO^+^ adduct, we used a collision cell (He collision gas) operating in a high-energy mode. The uncertainty of the measurement was less than 3%.

Antibacterial properties of PET and PET with immobilized AgNPs were tested by a standard drop plate method, using two environmental bacterial strains: Gram-negative *Escherichia coli* (*E. coli*, DBM 3138) and Gram-positive *Staphylococcus epidermidis* (*S. epidermidis*, DBM 2124). Inocula of both bacterial strains were cultivated in liquid Lauria–Bertani medium in an orbital shaker at 37 °C for 16 h. Then, inocula were diluted to turbidimetry corresponding to 1.5 McFarland standard and were serially diluted in sterile phosphate buffered saline (PBS). The final concentration of bacteria in solution was 1 × 10^3^–1 × 10^4^/mL. Triplicates of samples were immersed in 2 mL of bacterial solution and incubated in static conditions for 3 h at 24 °C. The aliquots of 5 × 25 μL from each vortexed sample were placed on pre-dried agar plates (*E. coli* on LB agar, *S. epidermidis* on PCA agar). After overnight incubation on agar plates, the number of colony-forming units (CFU) of each strain was counted. The experiments were accomplished under sterile conditions.

## 5. Conclusions and Future Perspectives

Electrochemically synthesized round-shaped silver nanoparticles (AgNPs) were immobilized on the thin surface layer of a set of polymer foils by the action of linearly polarized light from an excimer KrF laser. We have shown that this technique, free of any chemical mediators, enables us to enrich the ultra-thin surface layer of a broad spectrum of polymers with AgNPs. Particles are localized in a few tens of nm beneath the surface with the highest concentration on the surface area and their anchorage to the polymer is very strong. In the case of polymers absorbing radiation, the higher applied fluencies simultaneously transform their surface morphology into a highly rugged morphology. Simulations of absorbed light-to-heat conversion on the AgNPs proved that the excitation temperature together with forward-directed optical force is sufficient for the particles to penetrate into the thin surface layer of the examined semi-crystalline polymers. An essential point seems to be the right choice of laser operational wavelength, which should be close to but not directly equal to the maximum of the LSPR induced in Ag nanoparticles. A correctly chosen wavelength ensures both (i) ample scattering necessary to evoke forward directed optical force and (ii) sufficient residual beam intensity enabling the transformation of the surface morphology of those polymers which absorb the light at the used wavelength. The accuracy of our simulations predicting the temperature increase at the level of individual nanoparticles was confirmed at the macroscopic level, by measuring the temperature in the interaction volume of silver colloids, which provided values of the same order. Preliminary antimicrobial tests have shown that laser-immobilized AgNPs may open up new possibilities in the development of the next generation cell-conform antimicrobial coatings of polymeric materials, particularly capable of inhibiting biofilm formation.

## Figures and Tables

**Table 1:** Concentrations of silver Ag3d, carbon C1s, oxygen O1s, and nitrogen N1s (in at. %) in pristine and AgNP-immobilized polymers, derived from XPS analysis.

Polymer	Laser Fluence (mJ cm^−2^)	Element Concentration (at. %)			
		Ag	C	O	N
**PET**	0	-	86.4	13.6	-
	14	11.3	72.3	16.4	-
	22	13.5	70.9	15.6	-
**PEN**	0	-	77.8	22.2	-
	14	7.3	70.1	22.6	-
	22	7.5	72.2	20.3	-
**PEEK**	0	-	86.4	13.6	-
	14	11.3	72.3	16.4	-
	22	13.5	70.9	15.6	-
**PA 6,6**	0	-	75.0	12.5	12.5
	14	0.5	77.5	12.4	9.6
	22	3.6	73.4	12.5	10.5
**PI**	0	-	75.8	17.3	6.9
	14	8.8	68.9	18.9	5.4
	22	11.3	67.8	18.1	2.8

**Table 2:** Concentrations of silver Ag3d, carbon C1s, oxygen O1s, and nitrogen N1s (in at. %) in pristine and AgNP-immobilized polymers, derived from XPS analysis.

Polymer	laser Fluence (mJ cm^−2^)	Concentration of Ag ions (μg/L)	
		3 h	24 h
**PET**	14	0.36	0.56
	22	0.87	1.34
**PEN**	14	0.48	0.72
	22	0.57	0.84
**PEEK**	14	0.28	0.31
	22	0.45	0.66
**PA 6,6**	14	0.07	0.13
	22	0.14	0.19
**PI**	14	0.13	0.34
	22	0.20	0.44

**Table 3:** The literature data on Kapitza conductance.

Conductance (MW m^−2^ K^−1^)	Polymer	Crystal	Object	Ref.
69.3 ± 17.1	PVAc, Le phase	Si	film	[57]
74.9 ± 18.1	PVAc, Le phase	Si	film	[57]
80.8 ± 9.4	PVAc, Le phase	Au	film	[57]
93.6 ± 18.0	PVAc, Le phase	Au	film	[57]
30.0 ± 10.0	PMMA	Al_2_O_3_	nanoparticle	[58]
26.0 ± 13.0	PMMA	Al_2_O_3_	nanoparticle	[58]
59.0	PMMA	Si/Au	film	[59]
115.0	PMMA	Si/Ti/Au	film	[59]

## Data Availability

Not applicable.
